# An audit of antimicrobial prescribing by dental practitioners in the north east of England and Cumbria

**DOI:** 10.1186/s12903-018-0682-4

**Published:** 2018-12-10

**Authors:** A. Sturrock, D. Landes, T. Robson, L. Bird, A. Ojelabi, J. Ling

**Affiliations:** 1 Faculty of Health Sciences and Wellbeing, Sciences Complex, City Campus, Chester Road, University of Sunderland, Sunderland, SR1 3SD UK; 2 PHE North East, Newcastle Upon Tyne, UK; 3 Local Dental Network, Tees, Durham, Darlington UK; 4 Health Education North EastNewcastle Dental Hospital, Newcastle upon Tyne, UK

**Keywords:** Antibiotics, Audit, Dental, Prescribing

## Abstract

**Background:**

Inappropriate prescribing of antimicrobials is a significant threat to global public health. In England, approximately 5% of all antimicrobial items are prescribed by dentists, despite the limited indications for their use in the treatment of oral infections in published clinical guidelines. The objective of this study was to survey antimicrobial prescribing by dental practitioners in North East England and Cumbria, identify educational and training needs and develop a self-assessment tool that can be used for Continued Professional Development by individual practitioners.

**Methods:**

During October 2016, 275 dental practitioners used a standardised form to record anonymous information about patients who had been prescribed antimicrobials. Clinical information and prescribing details were compared against clinical guidelines published by the Faculty of General Dental Practitioners UK.

**Results:**

Dental practitioners provided data on 1893 antimicrobial prescriptions. There was documented evidence of systemic spread, such as pyrexia in 18% of patients. Dentists recorded patients’ pain (91.1% of patients), local lymph gland involvement (41.5%) gross diffuse swelling (55.5%) dysphagia (7.2%) and trismus (13.6%). Reasons for prescribing antimicrobials included patient expectations (25.8%), patient preference (24.8%), time pressures (10.9%), and patients uncooperative with other treatments (10.4%). The most commonly prescribed antimicrobials were amoxicillin, accounting for 61.2% of prescriptions, followed by metronidazole (29.9%). Most prescriptions for amoxicillin were for either 5 days (66.8%) or 7 days (29.6%) and most prescriptions for metronidazole were for a 5-day course (65.2%) or 7-day (18.6%) course.

**Conclusion:**

In most cases, when an antimicrobial was prescribed, practitioners used the correct choice of agents and usually prescribed these at the correct dose. However, some evidence of suboptimal prescribing practices when compared to the Faculty of General Dental Practitioner guidelines were identified. The audit has identified training needs across the region and aided the development of Continued Professional Development sessions. Further work to identify barriers and facilitators for improving antimicrobial prescribing and determining appropriate methods to improve clinical practice are required.

**Electronic supplementary material:**

The online version of this article (10.1186/s12903-018-0682-4) contains supplementary material, which is available to authorized users.

## Background

Antimicrobial resistance is a significant threat to global public health. The World Health Organisation has stressed the seriousness of this problem and their Global Report of Surveillance in 2014 (p. IX) concluded that ‘A post-antimicrobial-era in which common infections and minor injuries can kill – is a very real possibility for the 21^st^ century’ [1].

Antimicrobial stewardship is defined by the UK National Institute for Health and Care Excellence (NICE) as ‘an organisational or healthcare-system-wide approach to promoting and monitoring judicious use of antimicrobials to preserve their future effectiveness’ [2]. Therefore commissioners, providers and individual practitioners should embrace antimicrobial stewardship with the view to improving practice [2]. When prescribing antimicrobials, prescribers should follow local antimicrobial guidelines, where available, or national guidelines, such as the UK Faculty of General Dental Practitioners (FGDP) guidelines on prescribing the shortest effective course, the most appropriate dose and the route of administration for appropriate clinical indications [3].

The FGDP antimicrobial prescribing guidelines for general dental practitioners recommends that antimicrobials are only indicated for the following [3]:

- As an adjunct to the management of acute or chronic infection
- For the definitive management of active infective disease such as necrotising ulcerative gingivitis
- Where definitive treatment has to be delayed due to referral to a specialist service, examples include inability to establish drainage in an uncooperative patient who requires sedation or general anaesthesia for treatment, or a patient who needs to be treated in a hospital environment due to comorbidities.

Antibiotic consumption has increased by 6.5% over the past 4 years in England [4]. Prescribing is measured as the defined daily dose (DDD) of antimicrobials taken by an individual per 1000 inhabitants per day. Prescribing increased from 21.6 DDD per 1000 inhabitants per day in England in 2011 to 23 DDD per 1000 inhabitants per day in 2014 [4]. In 2014, the majority (66.6%) of dental prescriptions were used to treat infections and although dental prescribing accounts for only 0.5% of all items prescribed in England [4], dentists are responsible for 5% of all antimicrobial drug prescriptions [5]. The highest combined general practice, hospital and dentist usage in England in 2015 at 27.4 DDD per 1000 inhabitants per day was Merseyside. Antibiotic prescribing is also high in the North East of England, with the second highest DDD nationally (25.8) in Cumbria, Northumberland and Tyne & Wear and the third highest (25.3) in Durham, Darlington and Tees [5]. Antibiotic consumption was 26.5% higher in Cumbria, Northumberland and Tyne and Wear than in Leicestershire and Lincolnshire which had the lowest usage of antimicrobials at 20.4 DDD per 1000 inhabitants per day [5]. The potential reasons for the variations in regional prescribing practices are unclear, this may be due to socioeconomic factors, although studies have found that this can only partly explain differences in antimicrobial use [6].

Clinical audit can provide practitioners with a way of assessing their compliance with current prescribing guidelines and there is evidence that antimicrobial prescribing can be improved following clinical audit, leading to a reduction in inappropriate prescribing and prescribing errors [7–9]. A recent audit of dental antimicrobial prescribing in Wales provides a benchmark for assessing prescribing in the North East and Cumbria. The authors found that 21.8% of antimicrobial prescriptions did not meet the dose, frequency or duration advised in clinical guidelines [9]. The specific prescribing choices for each indication were not reported; therefore our study has been designed to further explore the antimicrobial choices for each clinical diagnosis and present detailed prescribing information to further support the literature in this field.

The aim of this study was to produce data on antimicrobial prescribing by dental practitioners in the North East of England and Cumbria.

## Methods

### Design

The study was designed to capture prospective data on antimicrobial prescribing by dental practitioners in the North East and Cumbria during the month of October 2016. Dentists were asked to record information on the next 10 consecutive antimicrobial prescriptions they gave to patients from the start of the census period. The purpose of the audit tool was to capture the prescribing practices of participants and not the frequency of prescribing The standard used in the audit was the Faculty of General Dental Practitioners antimicrobial guidelines [3]. Comparative data for dental prescriptions dispensed by pharmacies in the North East of England and Cumbria during the month of the audit were also provided by the NHS Business Service Authority (BSA).

### Population

The population consisted of all dentists (2318) on the National Health Service (NHS) performer list working under NHS contracts in the North East and Cumbria. Participants were identified via NHS England, who posted all study materials to the registered address of providers with a general dental services or community dental services contract in the North East and Cumbria. A reminder letter was sent half way through the audit period.

### Audit tool

The audit tool (see Additional file 1) consisted of two parts. The first section captured demographic information of participating dental practitioners. This information included gender, age, country of undergraduate study and area of current practice. Information regarding the size of their practice, Dental Foundation Training or General Professional Training practice status and the number of patients typically seen per day was also obtained. The audit tool was piloted in two practices before being finalised.

The second section of the audit tool captured information on 10 antimicrobial prescriptions. Participants were asked to record information on the next 10 consecutive antimicrobial prescriptions they gave to patients; if participants did not prescribe antimicrobials to 10 patients during the audit period (October 2016), they were asked to return the audit tool with the data from the patients for whom they did prescribe.

The following information was recorded for each antimicrobial prescription:

- Patient information – age and allergy status
- Diagnosis and features – clinical diagnosis and presenting features, such as pyrexia, pain, local lymph gland involvement, diffuse swelling, dysphagia, trismus
- Antibiotic prescribed – name, dose, quantity, frequency, duration
- Influencing factors – patient expectations, preferences, time pressures, patients condition, uncertain diagnosis, failure of anaesthesia, uncooperative patients and failure of other treatments

### Procedure

The audit tool was posted by NHS England to dental practices in the North East and Cumbria during September 2016. A covering letter was included, explaining the background and purpose of the audit, the letter explained that the audit was voluntary, but would provide participants with 3 h of Continued Professional Development (CPD) for completing and returning the audit. A reminder letter was sent to all providers 2 weeks into the audit period.

### Ethics

The project was approved by the University of Sunderland Research Ethics Committee.

### Analysis

Completed audit templates were returned to the University of Sunderland for data entry and analysis using SPSS.

## Results

Based on data received from NHS England, there are an estimated 2318 performers in the North East and Cumbria. A total of 275 dental practitioners returned data on the audit, with a response rate of 11.9%. Practitioners provided data on 1893 antimicrobial prescriptions.

### Participant demographics

Of the 275 participants, 56% were male and 37.1% female; the remainder did not state their gender. The vast majority (81.8%) of participants had undertaken their undergraduate dental training in the UK, with the remaining studying in Europe (7.3%), and outside of Europe (4%); 6.9% of participants did not provide information on where they studied. Most participants described themselves as general dental practitioners (88.4%) with several orthodontists (2.2%) and community dentists (0.7%), also participating. The area of practice was missing from 8% of audit returns.

The largest proportion of responses came from Tyne and Wear with 35.6%, with Northumbria (10.2%), showing the smallest return. These returns are broadly as expected given the relative sizes of the populations of each of these areas. Data were missing from 6.9% of participants and the region was uncertain from the responses in 0.7% of participants.

Participants were asked to provide information on the practice in which they worked, 32.2% stated they worked in a Vocational Training (VT) or General Professional Training (GPT) practice; both VT and GPT practices are approved sites for the post-qualification training period required for UK graduates in order to work in NHS practice [10]. The modal number of practitioners working at a practice was 3 (range 1–23). The majority of participants, 62.5%, reported that they saw 21–30 patients per day.

### Antibiotic prescribing

Data on a total of 1893 antimicrobial prescriptions were reported in the audit. The mean age of patients who were prescribed antimicrobials was 43.02 years (SD 19.67 years); patients’ ages ranged from 1 to 91 years.

Allergy information was recorded for the majority of patients (see Table 1); this information was missing for 92 patients. Most patients (85.1%) did not have a known antimicrobial allergy; the most frequent antimicrobial allergy reported by patients was for amoxicillin, for 7.3% of patients.

**Table 1:** Frequency of documented antibiotic allergies

Antibiotic	Frequency	Percent
Amoxicillin	135	7.1
Metronidazole	19	1
Phenoxymethylpenicillin	10	0.5
Erythromycin	6	0.3
Amoxicillin and Phenoxymethylpenicillin	3	0.2
Cefalexin	3	0.2
Amoxicillin and Erythromycin	2	0.1
Amoxicillin and Metronidazole	2	0.1
Amoxicillin and Ampicillin	2	0.1
Amoxicillin, Ampicillin, Phenoxymethylpenicillin	2	0.1
Ampicillin	2	0.1
Amoxicillin, Ampicillin, Phenoxymethylpenicillin, Co-amoxiclav	1	0.1
Phenoxymethylpenicillin and Metronidazole	1	0.1
Azithromycin	1	0.1
Clindamycin	1	0.1
No known antibiotic allergy	1611	85.1
No information provided	92	4.9
Total	1893	100

Antibiotics were prescribed for a range of indications. The most common indication was an acute dento-alveolar infection (43.2% of prescriptions), followed by pericoronitis (14.7%) and periodontal abscess (11.7%). The indication was described as ‘other’ in 3% of prescriptions with no further description given. Indication was missing from the remaining 2.9% of prescriptions (see Table 2).

**Table 2:** Frequency of reported clinical diagnosis

Clinical Diagnosis	Frequency	Percent
Acute dento-alveolar infection	817	43.2
Pericoronitis	279	14.7
Periodontal abscess	221	11.7
Dry socket	145	7.7
Chronic dento-alveolar infection	130	6.9
Acute Dental Pain/Pulpitis	62	3.3
Necrotising ulcerative gingivitis	58	3.1
Other	57	3
Prophylactic antibiotics	25	1.3
Acute sinusitis	13	0.7
Gingivitis	12	0.6
Acute dento-alveolar infection and Acute Dental Pain/Pulpitis	2	0.1
Acute dento-alveolar infection and Chronic dento-alveolar infection	2	0.1
Acute dento-alveolar infection and Periodontal abscess	2	0.1
Acute dento-alveolar infection and Dry socket	2	0.1
Periodontal abscess and Necrotising ulcerative gingivitis	2	0.1
Acute dento-alveolar infection and Gingivitis	1	0.1
Acute dento-alveolar infection and Acute sinusitis	1	0.1
Necrotising ulcerative gingivitis and Dry socket	1	0.1
Pericoronitis and others	1	0.1
Local anaesthetic complication	1	0.1
Stomatitis	1	0.1
Tonsillitis	1	0.1
Trauma	1	0.1
No information provided	56	3
Total	1893	100

There was documented evidence of systemic spread, such as pyrexia in 18% of patients. Pain was reported in 91.1% of patients. Local lymph gland involvement was evident in 41.5% of prescriptions, with gross diffuse swelling present in 55.5% of cases. Dysphagia and trismus was present in 7.2 and 13.6% of patients respectively.

The most commonly prescribed antimicrobials were amoxicillin, accounting for 61.2% of prescriptions, followed by metronidazole (29.9%). The vast majority of patients were prescribed only one antimicrobial. Where multiple antimicrobials were prescribed, the most frequent combination was amoxicillin and metronidazole (1.7%). The frequency of each antimicrobial prescribed is shown in Table 3.

**Table 3:** Frequency of each antimicrobial prescribed

Antibiotic	Frequency	Percent
Amoxicillin	1159	61.2
Metronidazole	566	29.9
Erythromycin	53	2.8
Amoxicillin and Metronidazole	33	1.7
Cefalexin	8	0.4
Clarithromycin	7	0.4
Clindamycin	5	0.3
Co-amoxiclav	5	0.3
Other (specify)	4	0.2
Phenoxymethylpenicillin	3	0.2
Ampicillin	2	0.1
Cefradine	2	0.1
Doxycycline	2	0.1
Azithromycin	1	0.1
Clindamycin and Metronidazole	1	0.1
Co-amoxiclav and Metronidazole	1	0.1
No information provided	41	2.2
Total	1893	100

Data for dental prescriptions dispensed by pharmacies in the North East of England and Cumbria during the month of the audit was provided by the NHS Business Service Authority (BSA) (Additional file 2). The number of prescriptions issued for each antimicrobial is shown in Table 4. The frequency of prescriptions for each antimicrobial closely matches the data from this audit.

**Table 4:** The number of dental antibiotic prescriptions issued by pharmacies in the North East of England and Cumbria (NHS BSA prescribing data October 2016)

Antibiotic	Number of Prescriptions	Percent of prescriptions
Amoxicillin	9336	68.82
Metronidazole	3631	26.77
Erythromycin	420	3.10
Phenoxymethylpenicillin	44	0.32
Co-amoxiclav	33	0.24
Clarithromycin	30	0.22
Clindamycin	23	0.17
Cefalexin	21	0.15
Doxycycline	14	0.10
Tetracycline	8	0.06
Oxytetracycline	3	0.02
Azithromycin	2	0.01
Total	13,565	100.00

### Amoxicillin

Amoxicillin was typically prescribed at a dose of 500 mg (78.5%) or 250 mg (16.8%) and usually three times daily (98.3%). Most prescriptions for amoxicillin were for either 5 days (66.8%) or 7 days (29.6%) duration; the mean duration was 5.61 days (SD 1.06 days).

### Metronidazole

Metronidazole was the second most frequent antimicrobial prescribed, with doses of 400 mg accounting for 53.8% and 200 mg, 42.6% of cases. Almost all (98.1%) of prescriptions were for three times daily dosing and the mean duration of course was 5.13 days (SD 1.14). Most prescriptions were for a 5-day course (65.2%), 7-day (18.6%) or 3-day (12.9%) course.

### Erythromycin

Erythromycin was the third most prescribed antimicrobial identified in the audit (2.8%), when prescribed it was usually given at a dose of 250 mg (60.4%) or 500 mg (32.1%). It was prescribed 3 times daily in 22.6% or 4 times daily in 75.5% of cases. Erythromycin was prescribed for a mean duration of 5.49 days (SD 1.01).

### Reasons for prescribing antimicrobials

The reasons for prescribing an antimicrobial was documented for 91.9% of patients (participants could document more than one reason if appropriate). Data were missing for 8.1% of patients. Table 5 illustrates the reasons for prescribing and the frequency of responses. The patient’s condition was the most frequent reason given for prescribing an antimicrobial and was a documented factor in 90.4% of cases. This was followed by patient expectations and patient preference, 25.8 and 24.8% of cases respectively. Time pressure (10.9%), and patients uncooperative with other treatment (10.4%), were also reported by participants.

**Table 5:** Reported reason for issuing antimicrobial prescription

Reason for prescribing	Responses		Percent of Cases
	Number	Percent	
Patient’s condition	1572	50.8%	90.4%
Patient expectations	448	14.5%	25.8%
Patient preference	432	13.9%	24.8%
Time pressures	190	6.1%	10.9%
Patient uncooperative with other treatment	181	5.8%	10.4%
Unclear/uncertain diagnosis	116	3.7%	6.7%
Failure of other treatment	111	3.6%	6.4%
Failure of anaesthesia	47	1.5%	2.7%
Total	3097	100%	178 .1%

### Acute dento-alveolar infections

According to the FGDP guidelines, the first choice treatment for this indication is amoxicillin which should be prescribed for adults and children over the age of 6 years at a dose of 500 mg 3 times daily for up to 5 days. We found that amoxicillin was the most frequently prescribed antimicrobial and that the duration of treatment was in most cases 5 days (68%), although a number of prescriptions (28.6%) were for 7 days treatment. A lower dose of 250 mg was prescribed in 13.9% of cases. Systemic spread, such a pyrexia (18%) or local lymph glad involvement (41.5%), were not always present when prescribing antimicrobials.

Metronidazole is the second choice agent for this indication and in patients over 10 years of age should be prescribed at a dose of 400 mg three times daily for up to 5 days. The duration of prescribing matched the guidelines in 68.5% of cases, but prescriptions for 7 days duration (20.7%) were relatively frequent.

### Periodontal abscess

Treatment recommendations for a periodontal abscess are the same as those for an acute dento-alveolar infection. The most frequently prescribed antimicrobial for this indication was amoxicillin, typically prescribed for 5 days (61.5%) duration, or 7 days, (37.6%). Where metronidazole was prescribed, it was usually for 5 days (68.5%), or 7 days (20.7%). In 10% of cases amoxicillin was prescribed at a lower dose of 250 mg.

### Pericoronitis

FGDP guidelines recommend that treatment of pericoronitis should follow local measures unless patients present with pyrexia, spreading infection, persistent swelling or trismus. Where an antimicrobial is recommended, metronidazole should be the first choice, at a dose of 400 mg three times daily (patients aged over 10 years) for up to 5 days. The most frequently prescribed antimicrobial for the treatment of pericoronitis was metronidazole, typically prescribed for 5 days, (69.8%) duration or 3 days (16.4%). Where amoxicillin was prescribed, it was usually for 5 days (61.8%) or 7 days (35.5%) duration.

### Chronic dento-alveolar infection

Where antimicrobial treatment is required the treatment should follow the guidance for the treatment of acute dento-alveolar infections. The most frequently prescribed antimicrobial for the treatment of a chronic dento-alveolar infection was amoxicillin, typically prescribed for 5 days duration (66.3%), or 7 days (30.5%), a lower dose of 250 mg of amoxicillin recorded on 16.7% of prescriptions. Where metronidazole was prescribed, it was usually for 5 days (60.9%) or 7 days, (34.8%) duration.

### Dry socket

The management of dry socket involves local measures; with antimicrobials not routinely recommended. Practitioners reported prescribing for this indication with 7.7% of all prescriptions recorded for this indication. The most frequently prescribed antimicrobial for the treatment of a dry socket was metronidazole, typically prescribed for 5 days duration (59.4%) or 7 days (24.6%). Where amoxicillin was prescribed, it was usually for 5 days (76.2%) or 7 days (20.6%) duration.

## Discussion

This antimicrobial audit has provided prospective data on the prescribing of antimicrobials by 275 dentists in the North East of England and Cumbria. The majority of the respondents were general dental practitioners and trained in the UK.

Amoxicillin was the most frequently prescribed antimicrobial, followed by metronidazole and then erythromycin. This pattern of prescribing is confirmed by the NHS Business Services Authority prescription data, the antimicrobial prescriptions dispensed by community pharmacies during October 2016 closely matches those reported in this audit [11]. Antibiotics such as co-amoxiclav, cephalosporins, clindamycin and quinolones, which do not appear on FGCP guidelines were prescribed infrequently, by participants in this audit.

The most common indication for antimicrobial prescribing was for the treatment of an acute dento-alveolar infection. Other common indications included pericoronitis, periodontal abscess, dry socket and chronic dento-alveolar infections. For conditions such as dry socket, routine use of antimicrobials is not recommended and therefore indicates a potential need for further education of practitioners.

For the treatment of acute-dento alveolar infections FGDP guidelines recommend that antimicrobials should only be prescribed as adjuncts to treatment and there should be evidence of systemic spread, pyrexia and local lymph gland involvement [3]. Results from this audit suggest that evidence of systemic spread, such a pyrexia or local lymph glad involvement were frequently absent when prescribing antimicrobials. This finding is similar to a recent study performed in Wales, where only 37.2% of patients prescribed antimicrobials had signs of spreading infection or systemic involvement [9].

The patient’s condition was the key determining factor in practitioners prescribing antimicrobials; however, patient expectations were also frequent considerations in treatment choice. Work has been carried out in primary care medical practices to help educate patients about the unnecessary use of antimicrobials and there is evidence that public campaigns can potentially contribute to more careful use of antimicrobials [12]; this could be seen as a tool for reducing expectations in relation to dental prescribing.

Other reasons such as patient preference, time pressure and a lack of co-operation with other treatment options by patients were also commonly reported; these findings are similar to those from a recent study in Wales [13]. These are considerations for prescribers and further work to explore these factors using qualitative methods, would be beneficial in gaining a deeper appreciation of the reasons why practitioners prescribe antimicrobials, as well as what informs their choice of antimicrobial.

Following competition of the audit the dental antimicrobial stewardship (AMS) toolkit was published [5], by the Dental Subgroup of Public Health England’s English surveillance programme for antimicrobial utilisation and resistance (ESPAUR), FGDP and British Dental Association (BDA). The toolkit produced provided an audit tool for antimicrobial prescribing and patient focused information such as information leaflets and posters [14].

### Limitations

The audit was voluntary and reflected in the low response rate, therefore, those participating may be the practitioners who are most engaged in CPD and prescribing education. Therefore, those practitioners who were less likely to be up to date with current prescribing trends, were less likely to have been captured during this process. The audit was based on self-reports of the assessment of patient presentations and symptoms and also required dental practitioners to record data on antimicrobials that they prescribed over the audit period, rather than reporting retrospective data, as such the potential for practitioners to alter their prescribing habits (or checking the guidelines) needs to be taken into consideration.

### Recommendations

Based on our results, we make several recommendations:

- Health Education England and the Local Dental Networks should highlight current guidance in relation to the local management of dental infections to practitioners, such as mail out of FGDP guidance to practices.
- Educational opportunities for practitioners, focusing on the recommended indications, duration of therapy and doses for antimicrobial prescriptions should be explored, for example, dissemination of guidance or CPD sessions.
- Opportunities to educate patients on antimicrobial use in dentistry should be reviewed. The posters produced as part of the AMS toolkit and patient information leaflet should be displayed in dental practices.
- Further research building on the existing literature exploring the underlying factors that lead to the prescribing of antimicrobials by dental practitioners should be considered. This could be achieved through qualitative research, utilising interviews or focus groups with dental practitioners.

## Conclusion

This antimicrobial audit has shown that in most cases, when an antimicrobial is prescribed, practitioners are using the correct choice of agents and usually prescribing these at the correct dose. However, we did find some evidence of suboptimal prescribing practices which indicate that there may be a need for further support and/or training for prescribers. Underlying reasons for antimicrobial prescribing have been identified, but a deeper understanding is required to explore this fully and identify barriers or facilitators to improve clinical practice. The audit has identified training needs across the region and the development of CPD sessions on appropriate prescribing. The ESPAUR toolkit published subsequently to our audit provides an audit tool for practitioners.

## Additional files

Additional file 1: Audit tool. Description – Audit tool used to collect data. (DOCX 263 kb)

Additional file 2: Table showing number and description of antimicrobial prescriptions from dental practices dispensed by pharmacies with contracts in the North East and Cumbria. Description – Data provided by NHS BSA. (DOCX 16 kb)

## Abbreviations

AMS: Dental Antimicrobial Stewardship
BDA: British Dental Association
BSA: Business Service Authority
CPD: Continued Professional Development
DDD: Defined Daily Dose
ESPAUR: English Surveillance Programme for Antimicrobial Utilisation and Resistance
FGDP: Faculty of General Dental Practitioners UK
GPT: General Professional Training
NHS: National Health Service
NICE: UK National Institute for Health and Care Excellence
VT: Vocational Training
