# Supplementary material for: An audit of antimicrobial prescribing by dental practitioners in the north east of England and Cumbria
Source: BMC Oral Health. 2018 Dec 10;18:206. doi: 10.1186/s12903-018-0682-4 (PMC6288968; doi:10.1186/s12903-018-0682-4)
Supplement: Supplementary file 1 — Audit tool. Description – Audit tool used to collect data. (DOCX 263 kb) [file 12903_2018_682_MOESM1_ESM.docx]

**Supplementary 1 - Audit Template**
