# Supplementary material for: An audit of antimicrobial prescribing by dental practitioners in the north east of England and Cumbria
Source: BMC Oral Health. 2018 Dec 10;18:206. doi: 10.1186/s12903-018-0682-4 (PMC6288968; doi:10.1186/s12903-018-0682-4)
Supplement: Supplementary file 2 — Table showing number and description of antimicrobial prescriptions from dental practices dispensed by pharmacies with contracts in the North East and Cumbria. Description – Data provided by NHS BSA. (DOCX 16 kb) [file 12903_2018_682_MOESM2_ESM.docx]

**Supplementary 2 – Table showing number and description of antibiotic prescriptions from dental practices dispensed by pharmacies with contracts in the North East and Cumbria**.

| **Description of antibiotic** | **Number of prescriptions issued** |
| --- | --- |
| Amoxicillin 125mg/1.25ml oral suspension paediatric (20) | 2 |
| Amoxicillin 125mg/5ml oral suspension (100) | 164 |
| Amoxicillin 125mg/5ml oral suspension sugar free (100) | 188 |
| Amoxicillin 125mg/5ml oral suspension sugar free [A A H Pharmaceuticals Ltd] (100) | 1 |
| Amoxicillin 250mg capsules (15) | 435 |
| Amoxicillin 250mg capsules (21) | 717 |
| Amoxicillin 250mg capsules [Alliance Healthcare (Distribution) Ltd] (21) | 3 |
| Amoxicillin 250mg/5ml oral suspension (100) | 221 |
| Amoxicillin 250mg/5ml oral suspension [A A H Pharmaceuticals Ltd] (100) | 1 |
| Amoxicillin 250mg/5ml oral suspension [Kent Pharmaceuticals Ltd] (100) | 1 |
| Amoxicillin 250mg/5ml oral suspension sugar free (100) | 282 |
| Amoxicillin 250mg/5ml oral suspension sugar free [A A H Pharmaceuticals Ltd] (100) | 2 |
| Amoxicillin 250mg/5ml oral suspension sugar free [Kent Pharmaceuticals Ltd] (100) | 1 |
| Amoxicillin 3g oral powder sachets sugar free (2) | 69 |
| Amoxicillin 500mg capsules (15) | 3134 |
| Amoxicillin 500mg capsules (21) | 4098 |
| Amoxicillin 500mg capsules [A A H Pharmaceuticals Ltd] (15) | 3 |
| Amoxicillin 500mg capsules [A A H Pharmaceuticals Ltd] (21) | 5 |
| Amoxicillin 500mg capsules [Almus Pharmaceuticals Ltd] (21) | 6 |
| Amoxicillin 500mg capsules [Kent Pharmaceuticals Ltd] (15) | 1 |
| Amoxicillin 500mg capsules [Teva UK Ltd] (21) | 1 |
| Amoxil 500mg capsules [GlaxoSmithKline UK Ltd] (21) | 1 |
| Augmentin 375mg tablets [GlaxoSmithKline UK Ltd] (21) | 1 |
| Azithromycin 250mg capsules (6) | 2 |
| Cefalexin 250mg capsules (28) | 3 |
| Cefalexin 250mg tablets (28) | 2 |
| Cefalexin 500mg capsules (21) | 12 |
| Cefalexin 500mg tablets (21) | 4 |
| Clarithromycin 250mg tablets (14) | 17 |
| Clarithromycin 250mg/5ml oral suspension (70) | 1 |
| Clarithromycin 500mg tablets (14) | 12 |
| Clindamycin 150mg capsules (24) | 23 |
| Co-amoxiclav 250mg/125mg tablets (21) | 31 |
| Co-amoxiclav 250mg/62mg/5ml oral suspension (100) | 1 |
| Doxycycline 100mg capsules (8) | 14 |
| Erythrocin 250 tablets [AMCo] (100) | 3 |
| Erythrocin 500 tablets [AMCo] (100) | 13 |
| Erythromycin 250mg gastro-resistant tablets (28) | 365 |
| Erythromycin 250mg gastro-resistant tablets [A A H Pharmaceuticals Ltd] (28) | 2 |
| Erythromycin 250mg gastro-resistant tablets [Medreich Plc] (28) | 1 |
| Erythromycin ethyl succinate 125mg/5ml oral suspension (100) | 7 |
| Erythromycin ethyl succinate 125mg/5ml oral suspension sugar free (100) | 5 |
| Erythromycin ethyl succinate 250mg/5ml oral suspension (100) | 8 |
| Erythromycin ethyl succinate 250mg/5ml oral suspension sugar free (100) | 4 |
| Erythromycin ethyl succinate 500mg tablets (28) | 5 |
| Erythromycin ethyl succinate 500mg/5ml oral suspension sugar free (140) | 1 |
| Erythromycin stearate 500mg tablets (100) | 4 |
| Erythroped A 500mg tablets [AMCo] (28) | 1 |
| Erythroped Forte SF 500mg/5ml oral suspension [AMCo] (140) | 1 |
| Metronidazole 200mg tablets (21) | 1439 |
| Metronidazole 200mg tablets [A A H Pharmaceuticals Ltd] (21) | 1 |
| Metronidazole 200mg tablets [Almus Pharmaceuticals Ltd] (21) | 1 |
| Metronidazole 200mg/5ml oral suspension (100) | 64 |
| Metronidazole 200mg/5ml oral suspension [A A H Pharmaceuticals Ltd] (100) | 2 |
| Metronidazole 200mg/5ml oral suspension [Zentiva] (100) | 1 |
| Metronidazole 400mg tablets (21) | 2116 |
| Metronidazole 400mg tablets [A A H Pharmaceuticals Ltd] (21) | 2 |
| Metronidazole 400mg tablets [Almus Pharmaceuticals Ltd] (21) | 1 |
| Metronidazole 500mg tablets (21) | 4 |
| Oxytetracycline 250mg tablets (28) | 3 |
| Phenoxymethylpenicillin 125mg/5ml oral solution (100) | 5 |
| Phenoxymethylpenicillin 125mg/5ml oral solution sugar free (100) | 3 |
| Phenoxymethylpenicillin 250mg tablets (28) | 35 |
| Phenoxymethylpenicillin 250mg/5ml oral solution (100) | 1 |
| Tetracycline 250mg tablets (28) | 8 |
| **Grand Total** | **13565** |
